# SARS‐CoV‐2 Activated Peripheral Blood Mononuclear Cells (PBMCs) Do Not Provoke Adverse Effects in Trophoblast Spheroids

**DOI:** 10.1111/aji.70039

**Published:** 2025-01-07

**Authors:** Humblenoble Stembridge Ayuk, Susanne Arnold, Arkadiusz Pierzchalski, Mario Bauer, Violeta Stojanovska, Ana Claudia Zenclussen

**Affiliations:** ^1^ Department of Environmental Immunology Helmholtz Centre for Environmental Research Leipzig Saxony Germany; ^2^ Saxon Incubator for Translational Research University of Leipzig Leipzig Saxony Germany; ^3^ German Center for Child and Adolescent Health (DZKJ) Partner Site Leipzig/Dresden Leipzig/Dresden Germany

**Keywords:** 3D cell culture, peripheral blood mononuclear cells (PBMCs), pregnancy, SARS‐CoV‐2, spheroids, trophoblast invasion, trophoblast viability

## Abstract

**Problem:**

Although it is still uncertain whether Severe Acute Respiratory Coronavirus (SARS‐CoV‐2) placental infection and vertical transmission occur, inflammation during early pregnancy can have devastating consequences for gestation itself and the growing fetus. If and how SARS‐CoV‐2‐specific immune cells negatively affect placenta functionality is still unknown.

**Method of study:**

We stimulated peripheral blood mononuclear cells (PBMCs) from women of reproductive age with SARS‐CoV‐2 peptides and cocultured them with trophoblast spheroids (HTR‐8/SVneo and JEG‐3) to dissect if SARS‐CoV‐2‐activated immune cells can interfere with trophoblast functionality. The activation and cytokine profile of the PBMCs were determined using multicolor flow cytometry. The functionality of trophoblast spheroids was assessed using microscopy, enzyme‐linked immunosorbent assay (ELISA), and RT‐qPCR.

**Results:**

SARS‐CoV‐2 S and M peptides significantly activated PBMCs (monocytes, NK cells, and T cells with memory subsets) and induced the upregulation of proinflammatory cytokines, such as IFNγ. The activated PBMCs did not impact the viability, growth rate, and invasion capabilities of trophoblast spheroids. Furthermore, the hormonal production of hCG by JEG‐3 spheroids was not compromised upon coculture with the activated PBMCs. mRNA transcript levels of genes involved in trophoblast spheroid functional pathways were also not dysregulated after coculture.

**Conclusions:**

Together, the findings of our in vitro coculture model, although not fully representative of in vivo conditions, strongly support the claim that the interaction of SARS‐CoV‐2‐activated peripheral blood immune cells with trophoblast cells at the fetal–maternal interface does not negatively affect trophoblast functionality. This goes in hand with the recommendation of vaccinating pregnant women in their first trimester.

## 1 Introduction

Severe Acute Respiratory Coronavirus (SARS‐CoV‐2), the causative agent of coronavirus disease 19 (COVID‐19), currently accounts for more than 774 million confirmed cases and over 7 million mortalities worldwide since the beginning of the pandemic [1]. The disease spectrum ranges from a self‐limiting upper respiratory tract infection to pneumonia, and acute respiratory distress syndrome (ARDS) with possibilities of cytokine storm or extrapulmonary multiorgan complications [2, 3]. In several COVID‐19 patients, complications in the gastrointestinal tract, kidneys, and blood vessels were reported [4, 5, 6] due to SARS‐CoV‐2‐associated immune responses. Notably, SARS‐CoV‐2 infection is of major concern in pregnancy, as physiological and immunological changes occur in pregnant women [7, 8, 9] that might make them susceptible to COVID‐19 pathogenesis. Accumulating shreds of evidence show that pregnant women with severe COVID‐19 are likely to develop pregnancy complications such as stillbirth, preterm birth, and preeclampsia [10, 11, 12, 13], especially in the third trimester of pregnancy [14].

Recently, it has been shown that maternal respiratory SARS‐CoV‐2 infection can trigger a distal inflammatory immune response in the placenta [15]. The placenta, a newly formed organ during pregnancy is crucial for fetal growth and development. Tightly controlled proliferation, invasion, and hormone production of the human trophoblast cells is essential for normal placenta development [16, 17, 18]. Because of its unique anatomy, maternal blood is directly and constantly in contact with trophoblast cells. This, in turn, means that maternal immune cells from a very early stage of pregnancy, engage in crosstalk with the placenta [19, 20, 21] and support the functionality of trophoblast cells [22, 23, 24]. However, perturbation of this immunological environment can, in turn, lead to an increased risk of pregnancy dysfunctions and impact on fetal development. For example, maternal Zika virus infection was shown to trigger a Th17 pro‐inflammatory immune response leading to Congenital Zika Syndrome (CZS) in the offspring [25]. This implies that apart from an active Zika virus infection of the placenta, pro‐inflammatory responses triggered by the virus can dysregulate the placenta and interfere with fetal development, which might also be the case for SARS‐CoV‐2 infection. Furthermore, current studies have also shown that maternal immune activation with local inflammatory immune responses at the fetal–maternal interface from other viral infections can lead to abnormal fetal outcomes and adverse neurological consequences in the offspring later in life [26, 27, 28, 29]. Hence, it is worth studying if and how specifically SARS‐CoV‐2‐activated immune cells affect the functionality of the placenta.

During pregnancy, the immune response has to quickly adapt to the presence of paternal antigens in the conceptus and initiate a tolerogenic immune response toward them [24] while staying aware and prepared for possible pathogens. Upon SARS‐CoV‐2 infection, T cells, NK cells, macrophages, and stromal cells are mainly involved in the proinflammatory responses at the fetal–maternal interface [15, 30], and their infiltration within the placental villi can be associated with the upregulation of TH1/TH2 and NK cell activation pathways [31]. Therefore, it is likely to hypothesize that the infiltration of SARS‐CoV‐2‐activated peripheral immune cells, together with their proinflammatory mediators into the villous space might dysregulate or interfere with vital placental physiological processes, such as trophoblast proliferation, invasion, hormone production, or pregnancy loss.

The design of studies aimed to understand how SARS‐CoV‐2‐activated immune cells impact placenta functionality is challenging. To understand if and how SARS‐CoV‐2‐activated immune cells affect placental functionality, we preferred to use a 3D cell coculture model of trophoblast spheroids and peripheral blood mononuclear cell (PBMCs). Initially, we used trophoblast spheroids as a model that more faithfully captures the complexity of the maternal–fetal interphase [32, 33]. In addition, we used SARS‐CoV‐2 peptides (S, N, M) and Zika virus M peptide (Zv) to assess virus‐induced T‐cell responses in PBMCs from healthy women of reproductive age. After confirming their activation and cytokine secretion profile, we examined their impact on the physiological characteristics of HTR‐8/SVneo and JEG‐3 spheroids: viability, growth rate, invasion capabilities, hormone production, and gene expression profile.

## 2 Materials and Methods

### 2.1 PBMCs Sample Collection

Peripheral blood samples from six women of reproductive age (22–44 years) were collected with the information regarding their COVID‐19 vaccination status, and the number of times infected/recovered. One participant was vaccinated four times, four were vaccinated three times, and one was vaccinated twice (all mRNA vaccines). Apart from one participant who was never vaccinated, the rest were infected or recovered at least once at different time points after vaccination (Table 1). Blood samples were obtained in BD Vacutainer ethylenediaminetetraacetic acid (EDTA) tubes (Cat: 367526, BD‐Plymouth, UK). Written informed consent was obtained from all subjects before sample collection.

**TABLE 1:** Participants’ age, type, and number of COVID‐19 vaccinations/boosters received, and the number of times infected with COVID‐19 or recovered from SARS‐CoV‐2 infection.

Age	Type and number of vaccination			Infection times/recovered
	BioNTech	Moderna	AstraZeneca	
33	2	—	—	2
38	1	2	—	1
22	3	—	—	—
40	2	1	—	2
24	4	—	—	2
44	2	—	1	1

The blood samples were centrifuged at 1000 rpm for 10 min, 4°C for plasma collection and storage at −80°C. PBMCs were isolated using gradient centrifugation on Ficoll‐Paque Plus (GE Healthcare, Chicago, IL, USA), according to the manufacturer's instructions. After isolation, PBMCs were washed twice with PBS and cryopreserved with freezing media consisting of 10% dimethyl sulfoxide (DMSO) and 90% fetal bovine serum (FBS) with 13 × 10^6^ cells per cryogenic vial. The vials were immediately placed in an isopropanol freezing container (e.g., Nalgene Mr. Frosty) and stored at −80°C overnight. For long‐term storage, the PBMCs were transferred to liquid nitrogen (−189°C).

### 2.2 Trophoblast Cell Lines and Generation of Spheroids

The human first‐trimester extravillous trophoblast cell line HTR‐8/SVneo was cultured in RPMI 1640 medium (Invitrogen, Karlsruhe, Germany) supplemented with 10 mmol/L HEPES, 1 mmol/L sodium pyruvate, and 100 nmol/L minimum essential medium (MEM) non‐essential amino acids (Invitrogen, Karlsruhe, Germany). The human choriocarcinoma cell line JEG‐3 was cultivated in Dulbecco's modified DMEM medium (DMEM; Invitrogen, Karlsruhe, Germany). Both media were supplemented with 10% FBS and 100 nM penicillin/streptomycin (Invitrogen, Karlsruhe, Germany). To obtain the spheroids, 1 × 10^3^ of HTR‐8/SVneo or JEG‐3 cells were seeded in 96‐well ultra‐low attachment plates (Corning, Kennebunk, ME, USA) and incubated for 3 days at 37°C and 5% CO_2_.

### 2.3 SARS‐CoV‐2 and Zika Peptide Pools

Commercially available SARS‐CoV‐2 peptides covering the entire spike protein (S) (PepTivator SARS‐CoV‐2 Prot_S Complete; #130‐127‐951), membrane protein (M) (PepTivator SARS‐CoV‐2 Prot_M; #130‐126‐702) nucleocapsid protein (PepTivator SARS‐CoV‐2 Prot_N; #130‐126‐698) and Zika glycoprotein M (PepTivator Glycoprotein M; #130‐114‐923, all from Miltenyi Biotec, CA, USA) were used. The lyophilized peptides were dissolved in sterile water (Ampuwa, PZN 04801694) according to the manufacturer's instructions, and a concentration of 2 µg/mL was used to stimulate PBMCs.

### 2.4 PBMC Stimulation With SARS‐CoV‐2 and Zika Virus Peptides

Cryopreserved PBMCs were rapidly thawed in a 37°C water bath and washed twice with RPMI 1640 medium. The cells were cultivated in RPMI 1640 medium supplemented with 10% FBS, 1% penicillin–streptomycin, 10 mmol/L HEPES, 1 mmol/L sodium pyruvate, and 100 nmol/L MEM non‐essential amino acids. 1 × 10^6^ cells were seeded per well (for immunophenotyping and spheroid coculture separately) in a U‐bottom 96 well plate (Greiner Bio‐One, Frickenhausen, Germany) overnight before stimulation. For stimulation of PBMCs, PepTivator SARS‐CoV‐2 (S, N, M) or PepTivator Zika virus M peptides were added to their respective culture wells at a final concentration of 2 µg/mL. Recombinant human IL‐2 (#200‐02‐50UG, PeproTech) and purified anti‐human CD28 (#302925, BioLegend) at concentrations of 30 and 6 µg/mL, respectively, were added to the peptide‐stimulated wells. Unstimulated PBMCs were used as negative controls. The Zika virus M peptide was used in this study as a control to observe the immune response toward an antigen to which the population was not exposed. The differences in the immune response between the Zika virus M peptide and the SARS‐CoV‐2 S, M, and N peptides attest that the SARS‐CoV‐2 recall responses are virus‐specific and not due to unspecific peptide interaction with immune cells. The plates were incubated for 6 and 24 h at 37°C and 5% CO_2_ with the protein transport inhibitor Brefeldin A (2 µg/mL) (# B‐7651, Sigma) added to the cell cultures (used for immunophenotyping) in the last 4 h of stimulation. After 6 and 24 h, the cells (for immunophenotyping) were transferred to a V‐bottom 96 well plate (Thermo Fisher Scientific Waltham, US) and centrifuged at 300 × *g* for 5 min at 4°C. The supernatants were collected and stored at −80°C, while the cell pellets were processed for immunophenotyping. 5 × 10^3^ cells (for coculture) were transferred to ultra‐low attachment plates for coculture with HTR‐8/SVneo and JEG‐3 spheroids.

### 2.5 PBMC Immunophenotyping After Peptide Stimulation

PBMC pellets were resuspended and washed with 1× PBS (without Ca^2+^, Mg^2+^, and 1% FBS). Live and dead cells were differentiated by staining with the Fixable Viability Dye eF506 (#65‐0866‐14; Invitrogen) for 15 min at 4°C in the dark. For surface staining, the cells were washed and incubated with fluorochrome‐conjugated antibodies (Table S1) for 30 min at room temperature (RT) in the dark. The cells were then washed, fixed in FACS BD Lysing solution (BD Biosciences, San Jose, USA) for 10 min at RT and permeabilized using FACS BD Perm2 solution (BD Biosciences, San Jose, USA) for 10 min at RT before staining the cells intracellularly (Table S1). After a further washing step, the cells were resuspended in 0.2 mL of wash buffer and measured using a CYTEK Aurora 3 Laser device (CYTEK Biosciences, Fremont, CA, USA). Data analysis and plotting of graphs were performed using FlowJo software version 10.8.1. For immunophenotyping, lymphocytes and non‐lymphocytes were identified among the total PBMCs using forward scatter area (FSC‐A) and side scatter area (SSC‐A). This was followed by the exclusion of doublets and dead cells, and the identification of immune cells such as monocytes, NK cells, and T cells. Specific subtypes were also further identified including monocyte subsets; classical monocytes (CD16^−^CD14^++^), intermediate monocytes (CD16^+^CD14^++^), and non‐classical monocytes (CD16^++^CD14^+^), NK cell subsets; CD56^bright^ (CD56^+++^CD16^+^) and CD56^dim^ (CD56^++^CD16^+^). For T cells, T helper CD4^+^ naïve cells (T_N_; CD3^+^ CD4^+^ CD45RA^+^CCR7^+^), effector memory (T_EM_; CD3^+^ CD4^+^CD45RA^−^ CCR7^−^), central memory (T_CM_; CD3^+^CD4^+^CD45RA^−^CCR7^+^), and T cytotoxic CD8^+^ naïve cells (T_N_; CD3^+^CD8^+^CD45RA^+^CCR7^+^), effector memory (T_EM_; CD3^+^ CD8^+^CD45RA^−^ CCR7^−^), central memory (T_CM_; CD3^+^CD8^+^CD45RA^−^CCR7^+^). The gating strategy is presented in Figure S1.

### 2.6 Trophoblast Spheroids Viability Assay

Spheroids (3 days post cell seeding as described above), were cocultured with PBMCs stimulated for either 6 or 24 h with SARS‐CoV‐2 peptides (S, N, M), and Zika virus M peptide (Zv), for 4 days. The spheroids were stained after 4 days of coculture to assess their viability. Staining was performed as previously described [57, 67]. Without fixation, trophoblast cells were stained with the following dyes at concentrations of 2 µM Calcein AM (# C1430), 4 µM Ethidium homodimer (# E1169), and 33 µM Hoechst 33342 (# H1399) (all from Invitrogen) for 3 h. To reduce the disturbance of the spheroids during the staining procedure, the dyes were added with precaution directly to the medium. Fluorescent images were taken after 3 h of incubation using a Keyence microscope with 10X Pan Fluor objective, and fluorescence intensity analysis was performed using ImageJ 1× software (National Institute of Health, Bethesda, MD, USA).

### 2.7 Spheroids Growth Rate Assay

When the 6 or 24 h SARS‐CoV‐2 peptides (S, N, M), and Zika virus M peptide (Zv) stimulated PBMCs were cocultured with HTR‐8/Svneo and JEG‐3 spheroids, brightfield images were taken at 0, 24, 48, and 96 h using the Keyence BZ‐X800 microscope with the 10X Pan Fluor objective. The growth area and diameter of the spheroids were calculated using the BZ‐X800 analyzer software.

### 2.8 Spheroids 3D Invasion Assay

After spheroid formation, cells were cocultured with stimulated PBMCs in 100 µL of the respective medium. Matrigel Basement Membrane Matrix, LDEV‐free (Corning, Bedford, MA, USA) at a concentration of 2.25 mg/mL dissolved in 100 µL complete medium (depending on the cell line used) was added on the side of each well and allowed to solidify for 30 min at 37°C and 5% CO_2_. Brightfield images of the spheroids during the invasion process were obtained at 0, 24, 48, 72, and 96 h post‐Matrigel embedding using a Keyence BZ‐X800 microscope with the 10X Pan Fluor objective.

### 2.9 Quantification of β‐HCG in Coculture Supernatants

Supernatants were collected 96 h after coculture of the stimulated PBMCs with spheroids and centrifuged for 10 min at 14 000 rpm at 4°C. The supernatants were stored at −80°C while awaiting analysis. Secretion of β‐HCG in the supernatant was quantified using a human β‐HCG enzyme‐linked immunosorbent assay (ELISA) kit (DRG, Marburg, Germany) following the manufacturer's instructions. The absorbance was measured using an Infinite F200 microplate reader (Tecan, Grödig, Austria).

### 2.10 mRNA Expression Using Quantitative RT‐PCR

Total RNA was extracted from eight spheroids using TRIzol (Life Technologies, Carlsbad, CA, USA) as stated in the manufacturer's protocol. RNA integrity and quantity were assessed using Infinite F200 NanoQuant (Tecan, Grödig, Austria). cDNA synthesis was performed with 0.6 µg RNA using the ImProm‐IITM Reverse Transcription System (Promega, Mannheim, Germany). Primers (Table 2) were selected by the Universal Probe Library Assay Design Center (http://qpcr.probefinder.com/organism.jsp (accessed until December 31, 2019). The RT‐PCR procedure and analysis were done as previously described [68]. The expression values of mRNA transcripts were determined by using the 2^−∆∆CT^ method with Ct values normalized to the geometric mean of two reference genes: GAPDH and ACTB.

**TABLE 2:** RT‐qPCR primers.

Gene	Forward primer	Reverse primer
*ACKR2*	GACTACGCACTCCAGGTAACAG	AAGCCTTCAGGTACTGGCGGAA
*ACTB*	GGACTTCGAGCAAGAGATGG	AGCACTGTGTTGGCGTACAG
*CASP3*	GGAAGCGAATCAATGGACTCTGG	GCATCGACATCTGTACCAGACC
*CDH2*	CCTGGATCGCGAGCAGATAG	CCAGGCTTTGATCCCTCAGG
*CDKN1A*	AGGTGGACCTGGAGACTCTCAG	TCCTCTTGGAGAAGATCAGCCG
*CDKN2A*	ACCAGAGGCAGTAACCATGC	CCTGTAGGACCTTCGGTGAC
*EGFR*	GTAAGAAGTGCGAAGGGCCT	AGTCACCCCTAAATGCCACC
*FGFR1*	GCACATCCAGTGGCTAAAGCAC	AGCACCTCCATCTCTTTGTCGG
*GAPDH*	GAGTCAACGGATTTGGTCGT	TTGATTTTGGAGGGATCTCG
*H2AFX*	CGGCAGTGCTGGAGTACCTCA	AGCTCCTCGTCGTTGCGGATG
*TNFRSF10D*	CTGCTGGTTCCAGTGAATGACG	TTTTCGGAGCCCACCAGTTGGT
*XRCC1*	CGGATGAGAACACGGACAGTGA	GAAGGCTGTGACGTATCGGATG

### 2.11 Statistical Analysis

Statistical data analysis was performed using the GraphPad Prism software (version 9.0; GraphPad Software, San Diego, CA, USA). The results were confirmed in three independent experiments, and all data are presented as mean ± SD. Differences between groups with normal distribution were calculated using one‐ or two‐way ANOVA followed by the Bonferroni multiple comparison test, while the Kruskal–Wallis test followed by Dunn's post‐hoc test was used for the non‐parametric test. Differences were considered statistically significant at *p* < 0.05.

## 3 Results

### 3.1 Characterization of Innate and Adaptive Immune Response of PBMCs After SARS‐CoV‐2 or Zika Virus Peptide Stimulation

**FIGURE 2:**
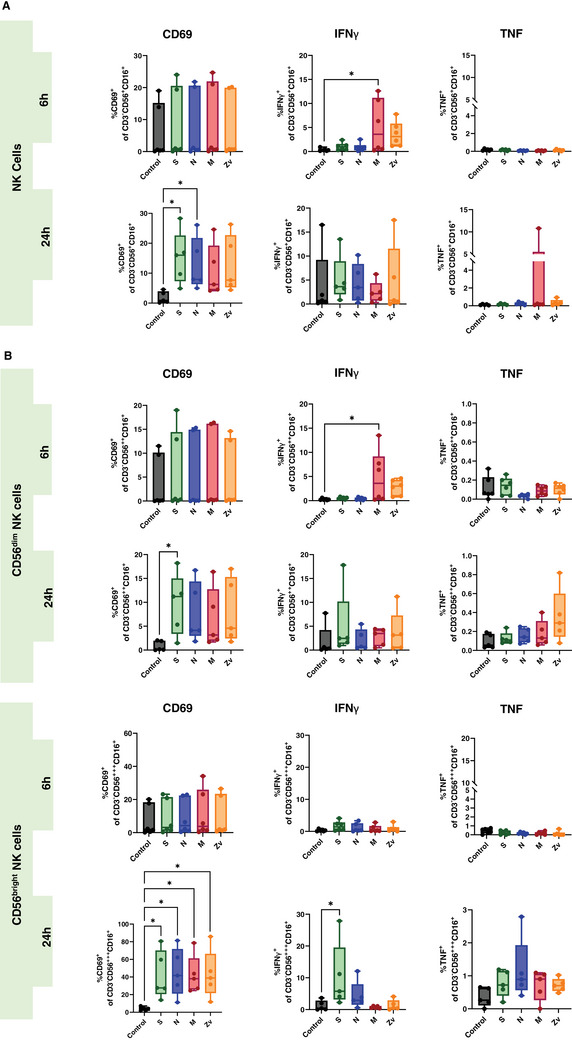
Natural killer (NK) cells response to SARS‐CoV‐2 (S, N, M) and Zika Virus M (Zv) peptide. PBMCs from non‐pregnant females were stimulated for 6 h (*n* = 5) or 24 h (*n* = 6) with SARS‐CoV‐2 S, N, M and Zika virus M peptide. Bar graph presentations of flow cytometry data to determine the general percentage of activation marker expression (A) in total NK cells and (B) in NK cell subtypes; CD56^bright^ (CD56^+++^CD16^+^) and CD56^dim^ (CD56^++^CD16^+^). *p < 0.05.

Ideally, exposure to a viral infection or vaccination should elicit a successful innate and adaptive immune response to specific viral components. Numerous studies have demonstrated the immune response to the SARS‐CoV‐2 live virus, as well as to its particles, proteins, and peptides in both vaccinated and convalescent individuals [34, 35, 36]. In this study, we concentrated on the in vitro innate and adaptive immune responses to PBMCs that were exposed to SARS‐CoV‐2 and Zika virus peptides from female participants. All participants were non‐pregnant females of reproductive age, all vaccinated against COVID‐19, with only one having never been infected with the virus (Table 1). PBMCs from these participants were stimulated for 6 or 24 h with SARS‐CoV‐2 peptides S, N, and M, as well as with the M peptide of Zika virus. Multicolor flow cytometry and immunophenotyping were performed to specifically identify monocytes, NK cells, and T cells together with their respective subtypes (Figure S1). As shown in Figure 1A, monocytes showed significant activation of CD69 and CD86 with S peptide and CD80 with M peptide after 24 h of stimulation. Monocytes were sub‐divided into three subsets: classical (CD16^−^CD14^++^) intermediate (CD16^+^CD14^++^), and non‐classical (CD16^++^CD14^+^) and mainly the intermediate monocyte subtype population was activated upon stimulation (Figure 1B). NK cells showed a significant increase in IFNγ at 6 h of stimulation with the SARS‐CoV‐2M peptide and activation of CD69 after 24 h stimulation with S, and N peptides (Figure 2A). Regarding the NK cell subtypes, CD56^dim^ NK cells had significant IFNγ upregulation after 6 h of SARS‐CoV‐2 M peptide stimulation and CD69 expression 24 h after stimulation from S peptide. CD56^bright^ NK cells showed significant CD69 activation (S, N, M, and Zv) and IFNγ production in S peptide only upon stimulation for 24 h (Figure 2B). Next, we investigated the adaptive immune response, particularly the T‐cell response. In CD4+ T cells, there was an increase in IFNγ after 6 h of M peptide stimulation, whereas after 24 h, the S peptide showed increased activation of CD69 and IFNγ upregulation (Figure 3A). Similarly, CD8+ T cells showed significant activation of CD69 and IFNγ (Figure 3A). For the viral‐specific memory T cells repertoire, all CD4+ T helper memory subtypes showed significant IFNγ production (from the M peptide) after 6 h (Figure 3B). After 24 h, CD69 was significantly expressed in naïve, central, and effector memory repertoires (Figure 3B). In CD8+ cytotoxic memory T cells, CD69 and IFNγ were substantially expressed in the central and effector memory cells after 24 h of stimulation with S peptide (Figure 3C). Taken together, our data demonstrate a proinflammatory recall response to SARS‐CoV‐2 peptide stimulation in PBMCs from COVID‐19 vaccinated or convalescent females, specifically targeting the spike and membrane proteins. We did not observe such an immune response towards Zika virus M protein. Our findings replicate the activation profiles of immune cells during acute SARS‐CoV‐2 infection. We then employed these activated cells to test their in vitro effects on trophoblast spheroids, aiming to understand the impact of SARS‐CoV‐2 infection on placental functionality in early pregnancy.

FIGURE 1Monocyte activation in response to SARS‐CoV‐2 (S, N, M) and Zika Virus M (Zv) peptide. PBMCs from non‐pregnant females were stimulated for 6 h (*n* = 5) and 24 h (*n* = 6) with SARS‐CoV‐2 S, N, M and Zika virus M peptide. Flow cytometry was performed to determine the general percentage of activation marker expression (A) in the general monocyte population and (B) in monocyte subsets; classical (CD16^−^CD14^++^), intermediate (CD16^+^CD14^++^), and non‐classical monocyte (CD16^++^CD14^+^) subtypes. *p < 0.05, **p < 0.01.

**Figure:**
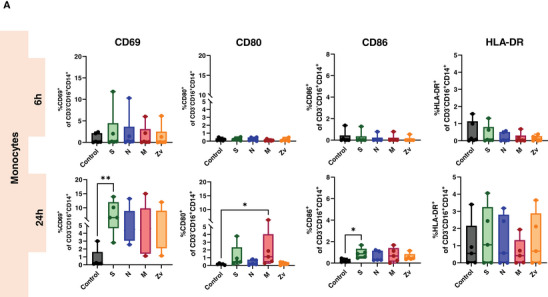

**Figure:**
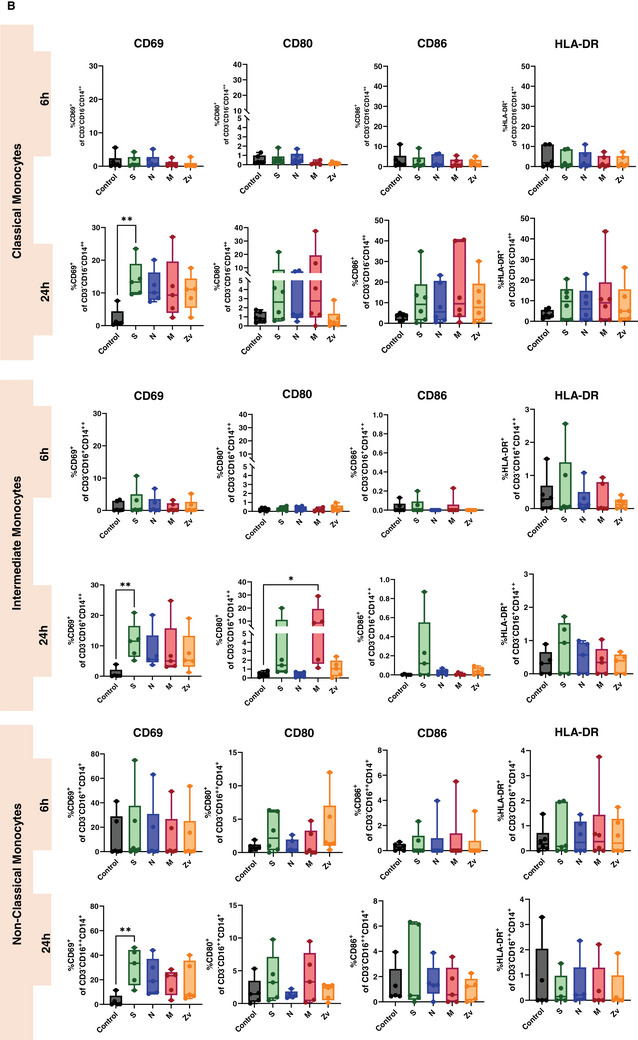

FIGURE 3T cells response to SARS‐CoV‐2 (S, N, M) and Zika Virus M (Zv) peptide stimulation. PBMCs from non‐pregnant females were stimulated for 6 h (*n* = 5) and 24 h (*n* = 6) with SARS‐CoV‐2 S, N, M and Zika virus M peptide. The percentage of activation markers expressing CD4+ and CD8+ T cells was determined by flow cytometry (A). Frequency of Circulating SARS‐CoV‐2 specific CD4+ memory cells (B) and memory CD8+ T cells (C). *p < 0.05, **p < 0.01.

**Figure:**
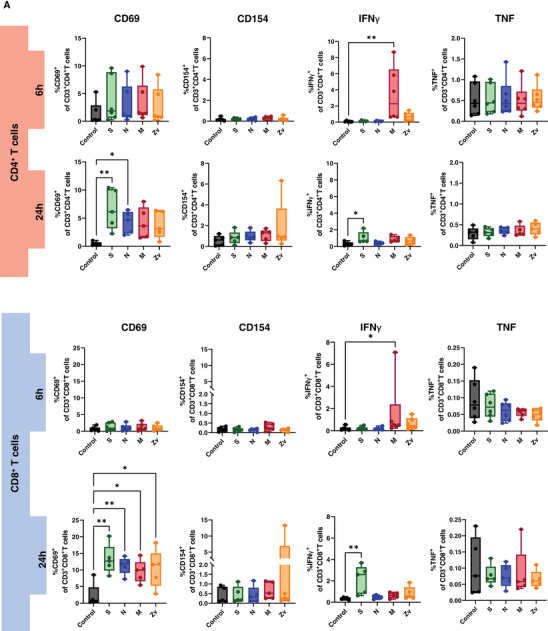

**Figure:**
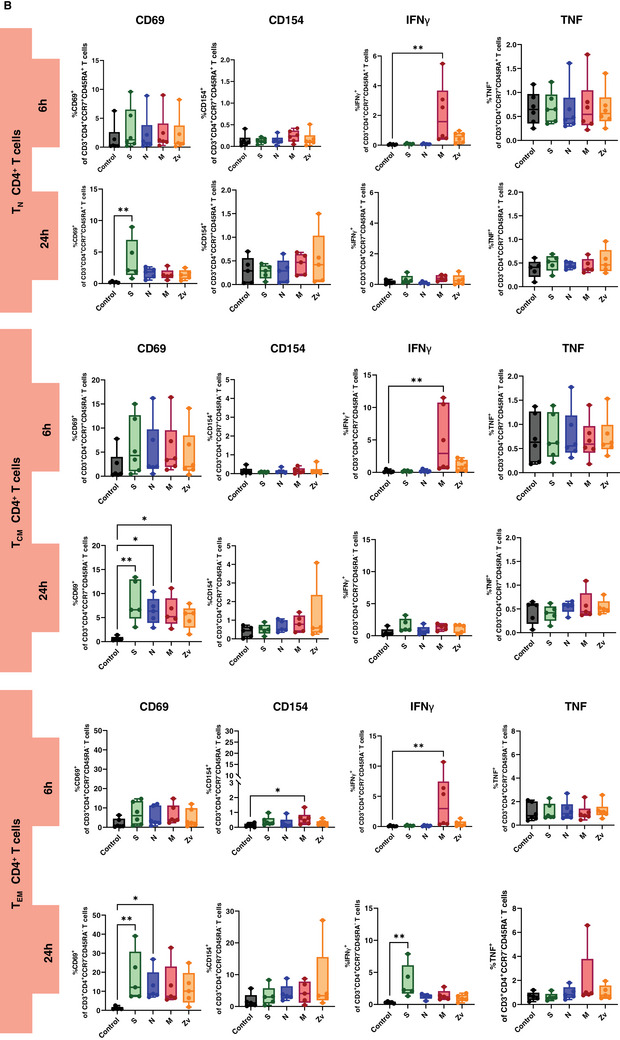

**Figure:**
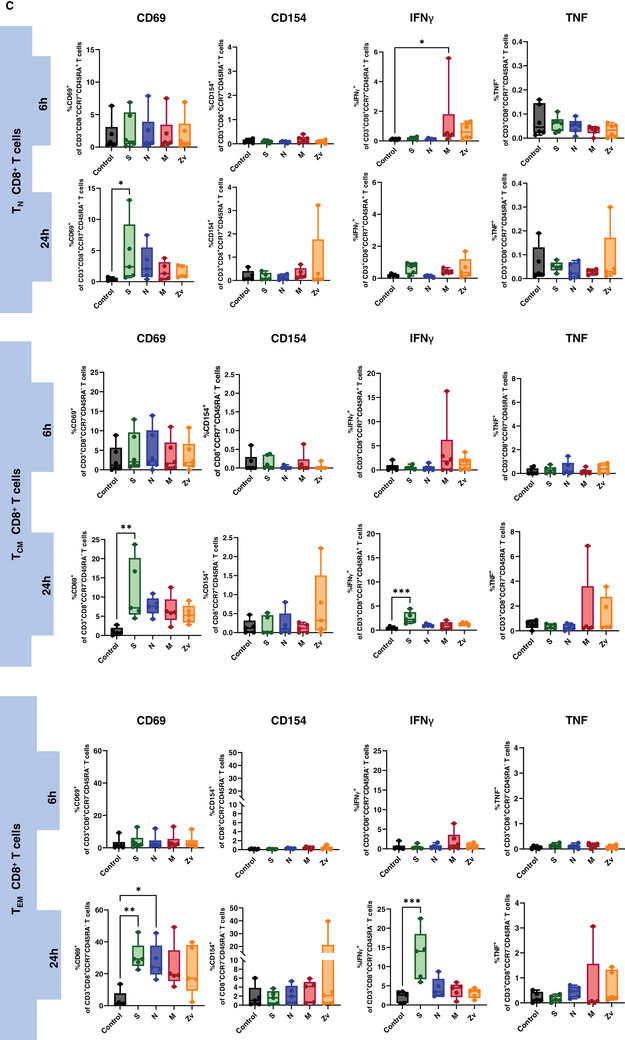

### 3.2 Viability of Trophoblast Spheroids After Coculture With Peptide‐Activated PBMCs

**FIGURE 4:**
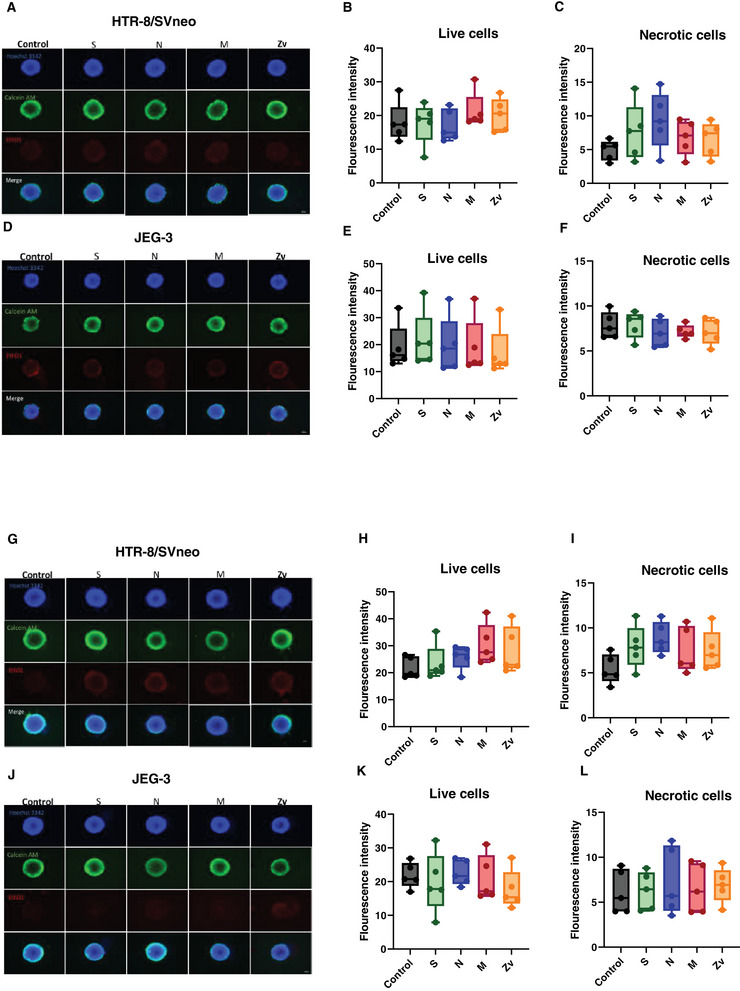
Viability of trophoblast cell line spheroids. Images from fluorescence microscopy showing the nuclei (blue), metabolically active cells (green), and necrotic cells (red) after 6 h (A, D) and 24 h (G, J) peptide stimulated PBMCs coculture with trophoblast spheroids for 4 days. Bar plots showing fluorescence intensities of live cells after 6 h (B, E) and 24 h (H, K) and necrotic cells after 6 h (C, F) and 24 h (I, L) of peptide stimulated PBMCs coculture. Scale bar 100 µm.

Considering the close interaction between maternal blood and trophoblast cells, there is a possibility that SARS‐CoV‐2‐activated peripheral cells may exert effects on trophoblast cells. First, we examined whether SARS‐CoV‐2 peptides (S, N, M) and Zika M (Zv) peptide‐stimulated peripheral mononuclear cells affect the viability of 3D trophoblast spheroids. We cocultured the 6 or 24 h peptide‐stimulated PBMCs and untreated PBMCs controls with HTR‐8/SVneo and JEG‐3 trophoblast spheroids for 4 days. As shown in (Figure 4A, B, D, E), 6 h peptide‐stimulated PBMCs showed no difference in the fluorescence intensity of active cells after 4 days of coculture with both HTR‐8/SVneo and JEG‐3 spheroids. Similarly, untreated PBMCs did not provoke any changes. This was also the case with necrotic cells (Figure 4A, C, D, F), as the fluorescence intensity was similar across all the peptides, and there was no difference compared to the unstimulated PBMCs control in both trophoblast spheroids. Similar results were obtained after 24 h coculturing experiments. There was no difference in the fluorescence intensity of active and necrotic cells in HTR‐8/Svneo (Figure 4G, H, I) and JEG‐3 (Figure 4J, K, L) spheroids after coculturing with either the peptide‐stimulated PBMCs or untreated controls. Together, these results show that the viability of trophoblast spheroids is not affected by the immune response triggered by SARS‐CoV‐2 peptide‐activated PBMCs.

### 3.3 HTR‐8/SVneo and JEG‐3 Spheroids Growth Rate After Coculture With Peptide‐Stimulated PBMCs

**FIGURE 5:**
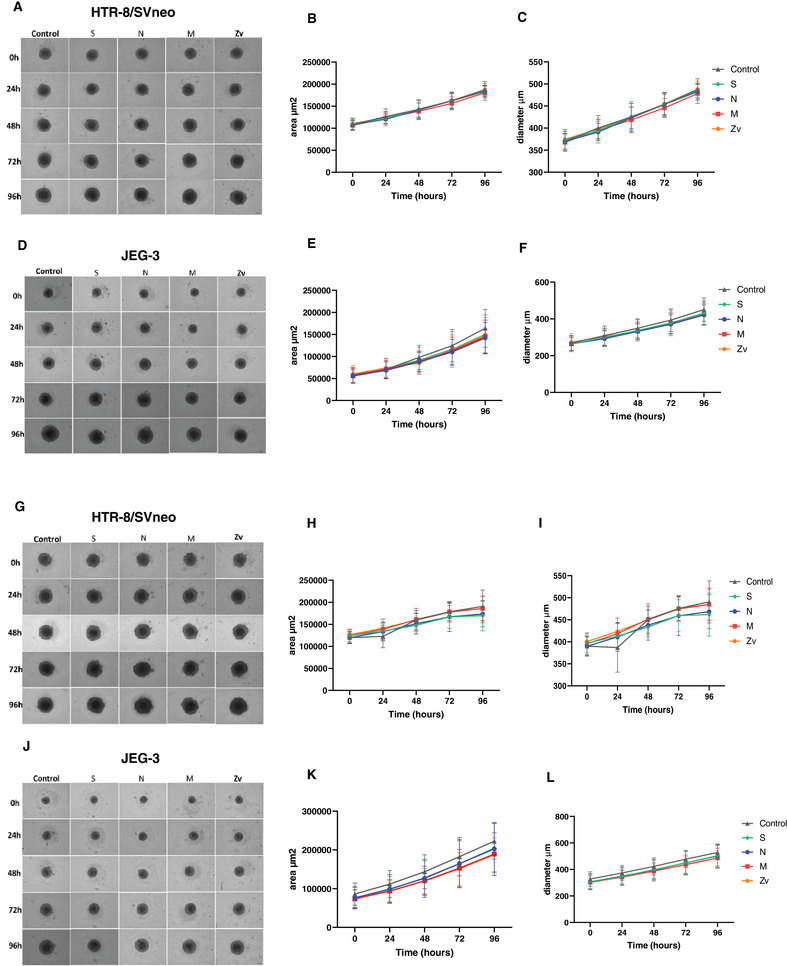
Growth rate of trophoblast spheroids. Bright‐field images of HTR‐8/Svneo and JEG‐3 spheroids after 6 h (A, D) and 24 h (G, J) peptide‐stimulated PBMC coculture for 4 days. Line graphs showing HTR‐8/Svneo spheroid growth area (µm^2^) and growth diameter (µm) after 6 h (B, C) and 24 h (H, I) and Line graphs showing JEG‐3 spheroid growth area (µm^2^) and growth diameter (µm) after 6 h (E, F) and 24 h (K, L) peptide stimulated PBMCs coculture. Scale bar 100 µm.

To assess whether the growth of HTR‐8/SVneo and JEG‐3 spheroids is altered due to the peptide‐stimulated PBMCs, we followed up the growth rate of each spheroid for 4 days. In HTR‐8/SVneo spheroids, 6 h peptide‐stimulated PBMCs did not affect the growth rate compared to the controls (Figure 5A). Furthermore, analysis of the mean spheroid area and diameter from all the peptide‐stimulated wells showed similar values compared to their respective controls (Figure 5B, C). This was also true for 24 h peptide stimulation (Figure 5G), as no changes in growth area and diameter were observed (Figure 5H, I). In JEG3 spheroids, no differences in spheroid growth were also observed either from 6 h (Figure 5D) or 24 h (Figure 5J) stimulation with the peptides during coculture. The mean spheroid area (Figure 5E, F) and diameter (Figure 5K, L) were similar among the wells with peptides‐activated PBMCs compared to the control. Together, these findings show that SARS‐CoV‐2 peptides‐activated PBMCs, do not perturb the normal growth trophoblast spheroids upon close interaction.

### 3.4 HTR‐8/SVneo and JEG‐3 Spheroids Invasion Rate After Coculture With Peptide‐Stimulated PBMCs

**FIGURE 6:**
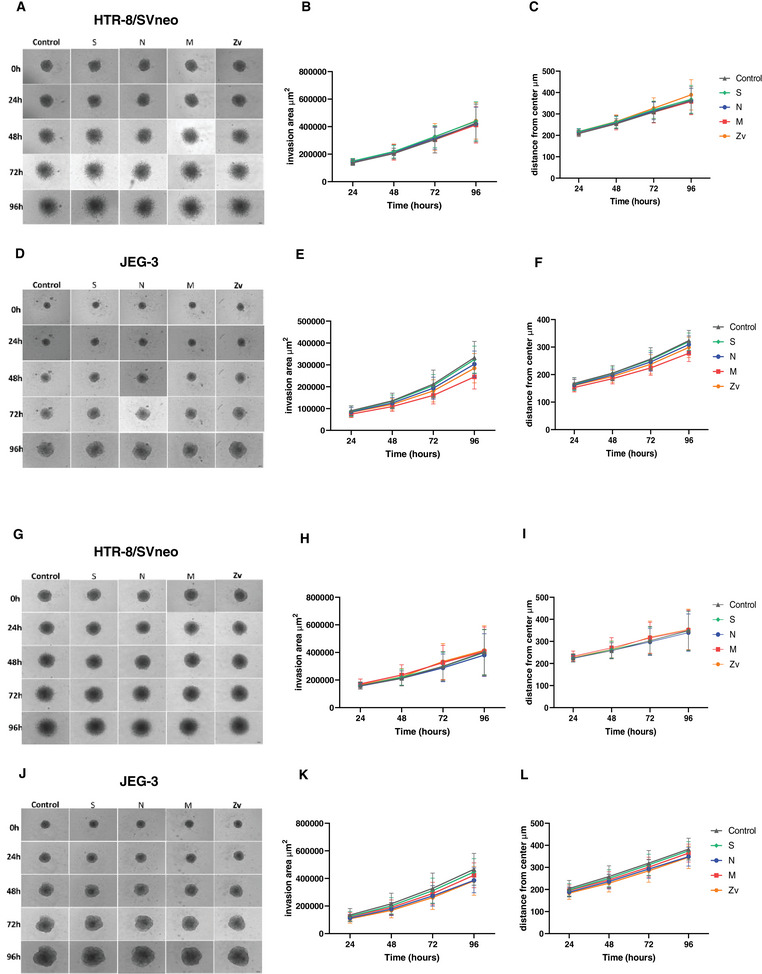
Invasion rate of trophoblast spheroids. Bright‐field images of HTR‐8/Svneo and JEG‐3 spheroids after 6 h (A, D) and 24 h (G, J) peptide‐stimulated PBMC coculture for 4 days. Line graphs showing HTR‐8/SVneo spheroid invasion area (µm^2^) and distance from the center (µm) after 6 h (B, C) and 24 h (H, I) and Line graphs showing JEG‐3 spheroid invasion area (µm^2^) and distance from the center (µm) after 6 h (E, F) and 24 h (K, L) peptide stimulated PBMCs coculture. Scale bar 100 µm.

To ensure proper implantation and optimal placentation that will lead to normal fetal growth and development, adequate invasion of the maternal endometrial stroma, and remodeling of the spiral arteries by trophoblast cells is necessary [37]. Next, we investigated the invasive potential of trophoblast spheroids upon interaction with SARS‐CoV‐2‐activated immune cells. To establish spontaneous invasion of the spheroids, they were cultured in Matrigel (gel‐like extracellular matrix) at a concentration of 2.25 mg/mL. The invasion was monitored for 4 days by observing and measuring invadopodia‐like projections from the spheroids. In HTR‐8/SVneo spheroids, radial protrusions were observed after 1 day of coculture with control PBMCs, 6 or 24 h SARS‐CoV‐2 peptide‐activated PBMCs and continued to increase in a uniform direction into the Matrigel over the 4 days of coculture (Figure 6A, G). Quantitative analysis of the total invasion area and invasion distance from the center showed no compromised invasion of the spheroids at 6 h (Figure 6B, C) or 24 h (Figure 6H, I). JEG‐3 spheroids, on the other hand, presented a bud‐like protrusion after coculture with activated immune cells or controls, which also kept growing over the 4 days of coculture (Figure 6D, J). Similarly, no statistical differences were observed among the invasive properties of the spheroids exposed to either 6 h (Figure 6E, F) or 24 h (Figure 6K, L) SARS‐CoV‐2 or Zika virus peptides stimulated PBMCs when compared to their unstimulated controls. Together, this data also indicates that the invasive capabilities of trophoblast spheroids are unaffected by their crosstalk with SARS‐CoV‐2‐activated PBMCs.

### 3.5 Quantification of Human Chorionic Gonadotropin (hCG) and mRNA Transcript Expression in Trophoblast Spheroids Upon Coculture With Peptide‐Stimulated PBMCs

**FIGURE 7:**
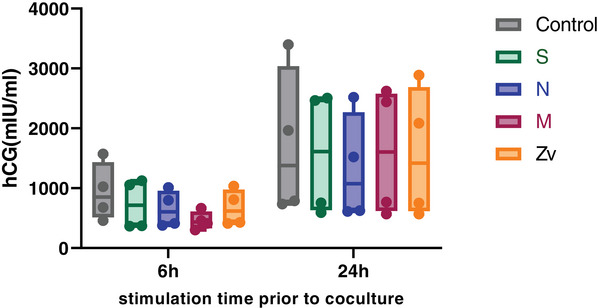
The effect of SARS‐CiV‐2 and Zika virus peptide‐activated peripheral blood mononuclear cells (PBMC) on human chorionic gonadotropin (hCG) secretion by JEG‐3 spheroids after 4 days of coculture.

Human chorionic gonadotropin (hCG) is one of the first embryonic signals detected during pregnancy and has immune regulatory functions [38]. Correspondingly, we investigated whether the amount of secreted hCG was modulated upon the interaction of trophoblasts with activated immune cells. Supernatants were obtained from JEG‐3 trophoblast spheroids after 4 days of coculture with 6 or 24 h SARS‐CoV‐2 or Zika virus peptide‐stimulated immune cells and unstimulated controls to checked for β‐hCG by ELISA. As shown in (Figure 7), there were no significant differences in β‐hCG secretion after 4 days of coculture with peptide‐stimulated PBMCs or control. There was also no significant difference when the 24 h stimulated peptides coculture was compared to the control. However, JEG‐3 spheroids cocultured with 24 h peptide‐stimulated or unstimulated PBMCs had higher β‐hCG secretion when compared to those of 6 h. Next, we investigated whether the co‐culturing of peptide‐stimulated PBMCs with trophoblasts spheroid will affect the gene expression profile of the trophoblast spheroids. We examine genes involved in the senescence pathway, DNA damage, apoptosis, cell adhesion, immune regulation, and cellular proliferation (Table 2). There were no significant changes in the mRNA expression levels in HTR‐8/SVneo spheroids after coculture with SARS‐CoV‐2 6 h peptide stimulated PBMCs (Figure S2A) or 24 h (Figure S2B) compared to the unstimulated controls. However, in JEG‐3 spheroids, both SARS‐CoV‐2 and Zika virus 24 h peptide stimulated PBMCs (Figure S2D) led to significant downregulation of TNFRSF10D mRNA levels after coculture.

## 4 Discussion

The direct impact of COVID‐19 on pregnancy outcomes has captivated global attention [39]. Despite some available cohort studies on SARS‐CoV‐2‐infected pregnant women, the dynamics of trophoblast SARS‐CoV‐2‐activated immune cell interaction is unknown. Due to the self‐limiting clinical course, unknown duration of SARS‐CoV‐2 viremia, and ethical constraints, studies including blood samples from COVID‐19 pregnant women at the acute phase remain challenging. Hence, we utilized PBMCs from healthy females in their reproductive age and exposed them to SARS‐CoV‐2 peptides to recapitulate viral‐related immune response. We studied the potential of SARS‐CoV‐2‐activated immune cells to affect trophoblast functionality. This is important, as several studies have shown that local inflammatory responses and immune cell activation at the placenta lead to pregnancy complications, such as fetal growth restriction, preeclampsia, and preterm birth [15, 40]. Moreover, irrespective of the absence of vertical transmission, maternal immune activation from viral infections at the placenta predisposes the fetus to neurological complications such as attention deficit hyperactivity disorder (ADHD), schizophrenia, microcephaly, and sensorial impairments later in life [41, 42, 43, 44].

In the first part of our study, we analyzed the SARS‐CoV‐2‐specific immune activation profile in PBMCs isolated from female participants, using unstimulated cells as negative controls and Zika virus M (Zv) peptide stimulation as a positive control. It was already known that patients presenting with COVID‐19 have been reported to have higher monocyte expansion and infiltration into bronchoalveolar fluids [45]. During pregnancy, monocytes are known to exhibit prominent antiviral functions [34, 46], and stimulation with SARS‐CoV‐2 particles leads to reactive oxygen species (ROS) production [30, 34]. We showed that stimulation with S and M peptides led to a significant upregulation of CD69 and CD80 expression in monocytes, particularly in intermediate monocytes. However, CD69 expression was also observed in classical and non‐classical monocytes. This result is in line with the study from Gomez‐Lopez et al. [34] where infection with SARS‐CoV‐2 virus led to the activation and expansion of monocytes. Furthermore, our observed increased activation of intermediate and non‐classical monocytes (known to be pro‐inflammatory) is in line with other studies in which intermediate monocytes [47] and non‐classical monocytes [48] were present in patients with mild and moderate COVID‐19 cases. Natural killer (NK) cells are potent innate immune antiviral cells that produce pro‐inflammatory cytokines such as IFNγ. They comprise two subtypes, CD56^bright^ and CD56^dim^, with immunoregulatory (in the decidua) and cytotoxic functions, respectively. We showed that stimulation with the SARS‐CoV‐2M peptide induced early production of IFNγ in CD56^dim^ NK cells, while the S peptide led to late IFNγ production in CD56^bright^ NK cells. These findings align with the results of Ustiuzhanina et al. [49] which showed that recovered COVID‐19 subjects had higher levels of IFNγ production in response to SARS‐CoV‐2 peptides by NK cells. Furthermore, a recent study [48] showed that COVID‐19 cases had a decrease in NK cell number which was compensated for by an increase in the number of IFNγ‐secreting cytotoxic NK cells. In accordance, our results showed substantial expression of IFNγ from peripheral CD56^bright^ NK cells which in contrast to their endometrial counterpart, are proinflammatory upon activation [50].

Next, we showed that stimulation of peripheral mononuclear cells from non‐pregnant females (recovered/vaccinated) with SARS‐CoV‐2 peptides, particularly S and M, leads to significant expression of the activation marker CD69 and upregulation of the proinflammatory cytokine IFNγ in both CD4+ and CD8+ T cells after 24 h of stimulation. Our findings are in agreement with other studies [35, 51] wherein stimulations with SARS‐CoV‐2 peptides and megapool peptides led to upregulation of activation‐induced markers in acute and convalescent COVID‐19 patients. In addition, Gomez‐Lopez et al. showed a similar T‐cell activation profile as well among pregnant women upon stimulation with SARS‐CoV‐2 particles and peptide pools [34]. Interestingly, we also observed the activation of virus‐specific memory T cell repertoire. Among CD4+ T cells, naïve (T_N_), central (T_CM_), and effector memory (T_EM_) cells triggered robust immune responses and were persistent within peripheral blood while among CD8+ cells, immune responses were mainly from central (T_CM_) and effector memory (T_EM_) cells. Our study is in agreement with other studies where memory T cell responses to SARS‐CoV‐2 peptide stimulation were not only associated with S protein but also with M and N among convalescent subjects [52, 53, 54, 55]. Thus, similarly to what we observed for innate immune cells among the PBMCs fraction, our in vitro activation model consisting of female PBMCs stimulated by specific viral peptides results in immune‐activated profiles of adaptive immune cells that resemble the activation profile of infected/vaccinated patients.

The implantation site during pregnancy encompasses crosstalk between the maternal peripheral blood and the fetal trophoblasts. We therefore cocultured virus‐specific activated immune cells with trophoblast‐derived spheroids. This was done to understand whether innate and adaptive immune changes resulting from viral infection or vaccination may alter trophoblast functionality, with a particular focus on features relevant to implantation and early pregnancy. We ran a battery of assays to understand the impact on trophoblast spheroid viability, growth, invasion properties, hCG secretion, and possible changes at the mRNA transcript level.

We showed that after co‐culturing trophoblast spheroids with peptide‐stimulated PBMCs, their viability was not affected. Our results are contrary to those of [56] where they observed that stimulated PBMCs (with IFNγ production) significantly decreased the viability of MDA‐MB‐231 spheroids, derived from breast carcinoma cell line. The absence of changes in spheroid viability confirms the validity of our in vitro model for studying additional features, effectively ruling out the possibility that the addition of activated immune cells inherently disrupts cell viability. Further, we investigated if the addition of SARS‐CoV‐2‐activated immune cells to the trophoblast spheroids may alter the normal growth rate of the spheroids. HTR‐8/SVneo and JEG‐3 spheroids can achieve a normal growth rate of up to 15 days in culture [57]. Here, we observed similar growth rates and showed that they were not perturbed upon coculture with SARS‐CoV‐2 or Zika virus 6 or 24 h peptide‐stimulated PBMCs for up to 4 days. Taken together, our results clearly show that the interplay between maternal SARS‐CoV‐2 experienced immune cells with trophoblast cells at the fetal–maternal interface does not affect the survival or physiological growth of the trophoblast cells.

To meet the demands of the growing conceptus, trophoblast invasion is required to remodel the maternal spiral arteries for sufficient maternal blood supply to the intervillous space to ensure normal fetal growth. Failure to achieve this has been associated with pregnancy‐related disorders, such as fetal growth restriction and preeclampsia [58, 59]. The current study showed that PBMCs from non‐pregnant females, SARS‐CoV‐2 recovered/vaccinated stimulated for 6 or 24 h with SARS‐CoV‐2 peptides or Zika virus peptide had no negative effect on the invasion rate of HTR‐8/SVneo and JEG‐3 spheroids, which were also similar to those reported in our previous study [33]. Therefore, irrespective of the viral activation profile and cytokine production (IFNγ) from the SARS‐CoV‐2 peptide‐stimulated PBMCs, the spheroids still invaded the extracellular matrix normally.

The early phase of pregnancy is characterized by the secretion of the hormone hCG from the implantation site. This hormone has been reported to immunoregulate peripheral immune cells at the fetal–maternal interface for efficient cross‐talk with trophoblast cells ensuring successful implantation and invasion [60, 61]. In line with previous findings [33], we also observed that JEG‐3 spheroids secreted hCG. Additionally, we observed no significant difference between the control and the respective peptide‐stimulated PBMCs after coculture. Furthermore, trophoblasts cocultured with PBMCs for 24 h had a higher hCG production after coculture. This might be due to the time difference before supernatant collection after coculture as more cells produce hCG when spheroids undergo cell division and growth. To understand if the activated PBMCs led to subtle changes in trophoblast spheroids at the transcriptomic level, we analyzed the mRNA transcripts of several genes important in senescence, DNA damage, apoptosis, cell adhesion, immune regulation, and cellular proliferation. There were no significant changes in the mRNA expression levels in HTR‐8/SVneo spheroids when the SARS‐CoV‐2 peptide‐stimulated PBMCs were compared to the unstimulated controls. In JEG‐3 spheroids, there was a significant downregulation of TNFRSF10D mRNA levels after coculture with SARS‐CoV‐2 24 h peptide stimulated PBMCs. TNF receptor superfamily member 10D (TNFRSF10D) also known as DCR2, is involved in the extrinsic apoptotic pathway [62]. However, its downregulation did not affect the viability of JEG‐3 trophoblast spheroids as already mentioned.

Overall, our data strongly concur with the results from one of the largest population‐based cohort studies published by Fallach et al. [63], wherein no increased risks of preterm birth and fetal growth restriction were observed for patients infected during the first trimester of pregnancy. Furthermore, other studies investigating pregnancy outcomes after COVID‐19 vaccination showed no predisposition towards pregnancy complications among SARS‐CoV‐2 vaccinated pregnant women compared to their unvaccinated counterparts [64, 65].

## 5 Conclusion

In summary, the in vitro model used in this study is the first to evaluate trophoblast functionality upon interaction with SARS‐CoV‐2 or Zika virus peptides‐activated innate and adaptive immune cells. Overall, we demonstrated that SARS‐CoV‐2 peptides (particularly S and M) activated PBMCs induced an immune profile comparable to the activation profiles observed upon infection and vaccination even among pregnant individuals. Of note, the magnitude of the innate and adaptive immune responses observed in the general population might show heterogeneity, with some individuals responding more robustly to viral activation than others. Nevertheless, our focus was to recapitulate the normal response of the general population, particularly, of females to understand how SARS‐CoV‐2‐activated PBMCs impact trophoblast cells. However, we showed that activated PBMCs had no effects on the functionality of HTR‐8/Svneo or JEG‐3 spheroids in terms of viability, growth, invasion, hormone production, and gene expression. Our in vitro findings support the use of SARS‐CoV‐2 vaccines during pregnancy and may partly explain why COVID‐19‐vaccinated pregnant women did not experience increased risks of pregnancy complications [66].

### 5.1 Limitations of the Study

The current study had several limitations. First, we lacked a PBMC group from pregnant women to compare the differential immune response kinetics in both groups and how they influence the physiology of trophoblast cells. However, current literature points to very similar immune responses upon infection [34, 63]. Secondly, the sample size of six participants used in this study is small and may affect the generalizability of our findings. We also acknowledge that variability in the immune responses observed among participants in our study could have also arisen from differences in the vaccination regimens, number of vaccinations, and number of times they were infected. Lastly, the 3D trophoblast spheroid in vitro coculture model used in this study might not faithfully recapitulate the complex interaction in vivo at the fetal–maternal interface; however, it serves as a useful alternative to study trophoblast functionality in vitro.

## Ethics Statement

All experimental protocols were approved by the Ethics Board at the University of Magdeburg (Study 28/08 and Study 179/18).

## Conflicts of Interest

The authors declare no conflict of interest.

## Supporting information



Supplementary materials

## Data Availability

The data that support the findings of this study are available from the corresponding author upon reasonable request.
