# Supplementary figures and images for: SARS‐CoV‐2 Activated Peripheral Blood Mononuclear Cells (PBMCs) Do Not Provoke Adverse Effects in Trophoblast Spheroids
Source: Am J Reprod Immunol. 2025 Jan 7;93(1):e70039. doi: 10.1111/aji.70039 (PMC11706221; doi:10.1111/aji.70039)

**Figure S1**

## Gating Strategy

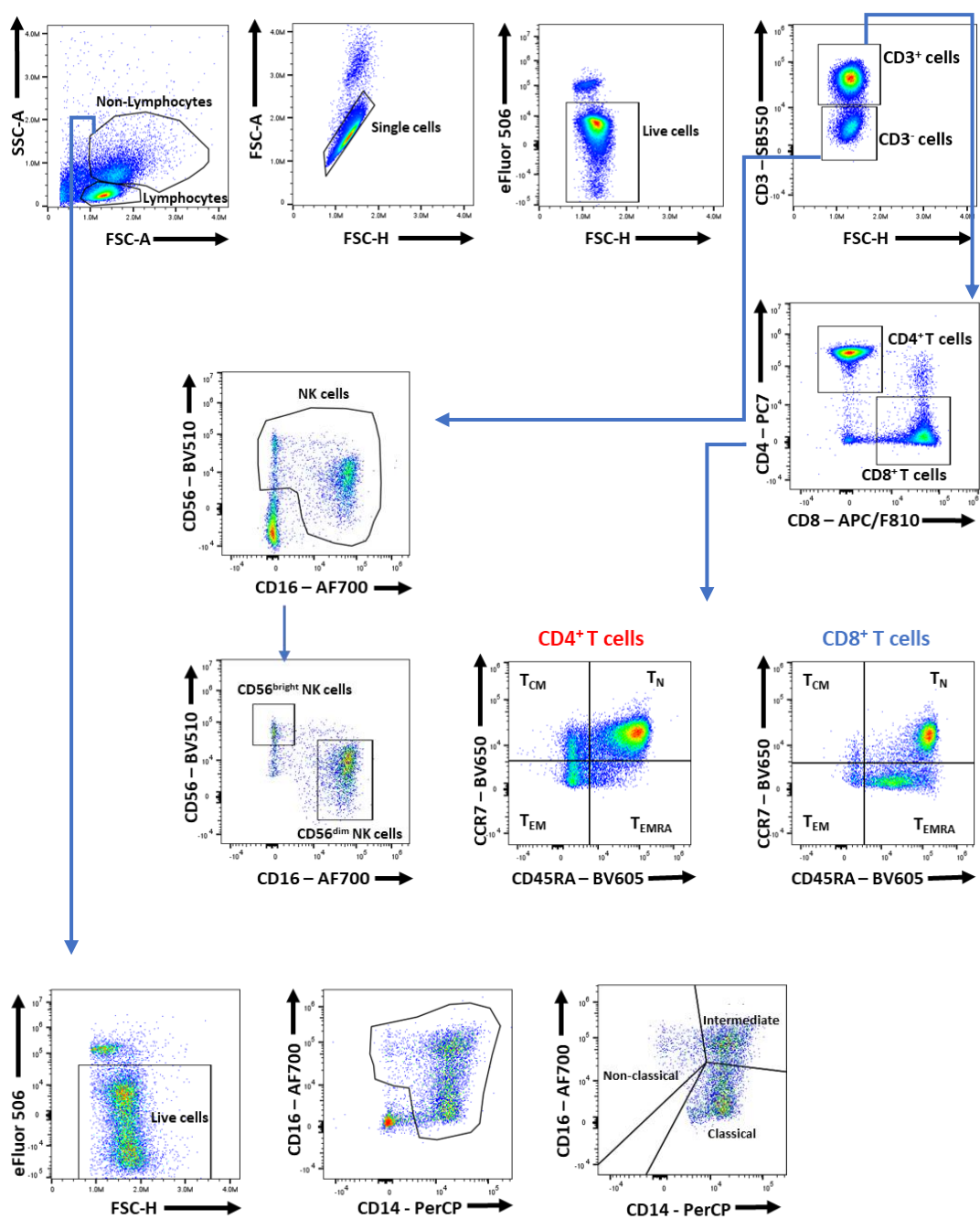

Figure S2

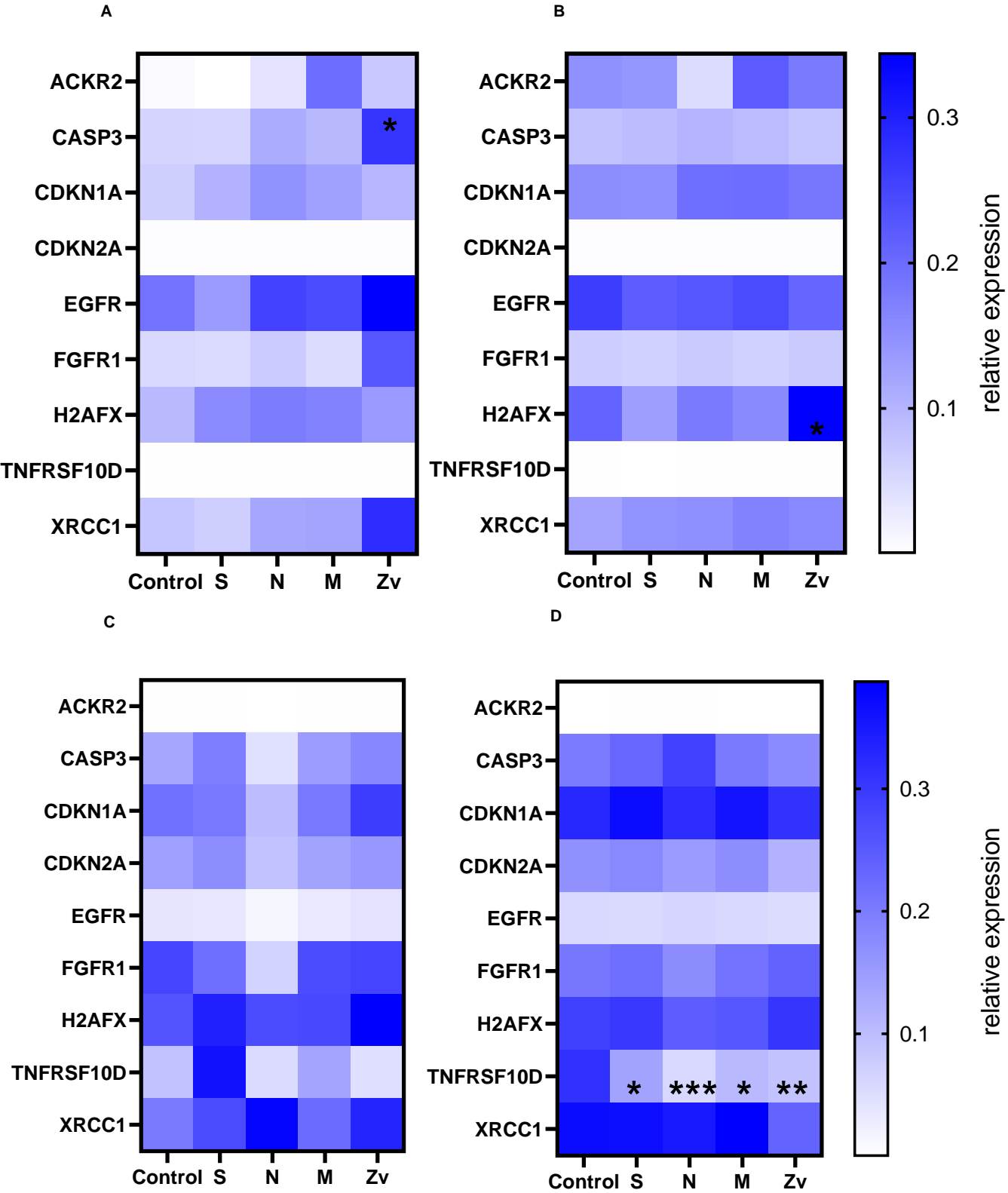

Supplement: Supplementary file 1 — Supplementary materials [file AJI-93-e70039-s001.pdf]
